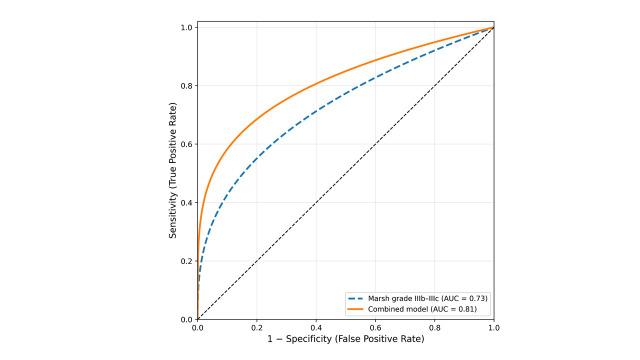# Clinical characteristics and associated factors of celiac disease complicated by *Helicobacter pylori*-negative chronic gastritis: A retrospective study

**DOI:** 10.1097/MD.0000000000048950

**Published:** 2026-05-29

**Authors:** Nan Gu, Li Ma, Lei Zhang, Fei-Ran Jia, Xiao-Yun Zhou, Yue Wang

**Affiliations:** a Department of Critical Medicine, Hebei Province Second Rongjun Youfu Hospital, Shijiazhuang, China; b Department of Internal Medicine, No. 8 Hospital of Shijiazhuang, Shijiazhuang, Hebei, China; c Department of Color Ultrasound, People’s Hospital of Yanshan County, Cangzhou, Hebei, China; d Laboratory of Biochemistry and Immunology, Cape Medical Laboratory, Shijiazhuang, Hebei, China.

**Keywords:** celiac disease, clinical characteristics, *Helicobacter pylori*-negative chronic gastritis, logistic regression, Marsh classification, receiver operating characteristic curve

## Abstract

Celiac disease (CD) is a chronic immune-mediated enteropathy characterized by gluten-induced small intestinal mucosal injury and a broad spectrum of clinical manifestations. Emerging evidence suggests that CD may also be associated with gastric mucosal abnormalities, including *Helicobacter pylori*-negative chronic gastritis (HPNCG). This study aimed to investigate the clinical, laboratory, and histopathological features of patients with CD complicated by HPNCG and to identify factors associated with this condition. A total of 60 patients with CD were retrospectively enrolled and divided into 2 groups: CD alone (n = 26) and CD with HPNCG (n = 34). Demographic characteristics, gastrointestinal symptoms, comorbidities, laboratory parameters, and histopathological findings were compared between groups. Univariate and multivariate logistic regression analyses were performed to identify factors independently associated with CD complicated by HPNCG. Receiver operating characteristic curve analysis was used to evaluate discriminatory performance. Among the 60 patients, 45.0% were male and 55.0% were female, with a mean age of 41.8 ± 15.2 years. Compared with the CD-alone group, patients with CD and HPNCG had significantly higher prevalence of abdominal bloating (61.8% vs 30.8%, *P* = .018), anemia (58.8% vs 26.9%, *P* = .013), osteopenia/osteoporosis (38.2% vs 15.4%, *P* = .048), and liver function abnormalities (23.5% vs 3.8%, *P* = .036). Histopathologically, Marsh grade IIIb–IIIc (88.2% vs 61.5%, *P* = .019) and moderate-to-severe chronic gastric inflammatory infiltration (64.7% vs 34.6%, *P* = .022) were more frequent in the CD with HPNCG group. Multivariate logistic regression identified Marsh grade IIIb–IIIc as the only factor independently associated with CD complicated by HPNCG (odds ratio = 3.96, 95% confidence interval: 1.05–14.91, *P* = .042). Receiver operating characteristic analysis showed that Marsh grade IIIb–IIIc alone yielded an area under the curve of 0.73, while the combined model achieved an area under the curve of 0.81. Patients with CD complicated by HPNCG present with a heavier burden of gastrointestinal symptoms, nutritional impairments, and mucosal injury compared to those with CD alone. Severe duodenal villous atrophy, reflected by Marsh grade IIIb–IIIc, is independently associated with HPNCG in CD and may serve as a clinically useful indicator for identifying this high-risk subgroup.

## 1. Introduction

Celiac disease (CD) is a chronic immune-mediated disorder triggered by gluten exposure in genetically susceptible individuals.^[1–3]^ It is primarily characterized by small intestinal mucosal injury, with villous atrophy, crypt hyperplasia, and inflammatory cell infiltration as the main histopathological features.^[4,5]^ These abnormalities impair nutrient absorption and may lead to a wide range of clinical consequences.^[6,7]^ Although CD affects approximately 1% of the general population worldwide, its recognition in clinical practice remains challenging.^[8,9]^

In addition to typical gastrointestinal manifestations, such as diarrhea, abdominal bloating, and weight loss, patients may present with extraintestinal features, including anemia, osteopenia or osteoporosis, and abnormal liver function.^[10–12]^ Although CD has traditionally been viewed as a disorder primarily involving small intestinal mucosal injury and malabsorption, growing evidence suggests that the inflammatory and immune abnormalities associated with CD may affect a broader extent of the gastrointestinal tract.^[9,13,14]^ Some patients with CD have been reported to present with chronic gastritis, increased inflammatory cell infiltration in the gastric mucosa, and other gastric histopathological changes.^[15–17]^ These findings suggest that gastric abnormalities may represent part of the broader spectrum of mucosal injury in CD.^[18,19]^ Such gastric changes may also contribute to a greater burden of digestive symptoms, nutritional impairment, and overall clinical complexity.^[20,21]^

*Helicobacter pylori*-negative chronic gastritis (HPNCG) refers to chronic inflammatory changes in the gastric mucosa in the absence of evidence of *H pylori* infection.^[22,23]^ Unlike classical *H pylori*-related gastritis, HPNCG likely arises from a more complex background and may be associated with immune dysregulation, medication exposure, other infectious factors, or alterations in the gastric mucosal environment.^[24,25]^ Some studies have reported that a higher prevalence of HPNCG in patients with CD, and chronic gastric inflammation may coexist with more severe small intestinal mucosal damage.^[21,22,24]^

Even so, the available evidence remains limited, and the findings have not been entirely consistent.^[24,26]^ Most previous studies have focused on the diagnosis of CD and the assessment of duodenal pathology,^[27–30]^ whereas the gastric manifestations of CD, particularly HPNCG, have received much less attention. As a result, the clinical profile of patients with CD complicated by HPNCG, including their symptoms, nutritional status, comorbidities, and histopathological features, has not been fully characterized. Based on these considerations, this study aimed to characterize the clinical, laboratory, and histopathological features of patients with CD complicated by HPNCG, and to identify factors associated with this condition. The findings may improve the clinical recognition of this subgroup and provide evidence to support more comprehensive evaluation and management in patients with CD.

## 2. Methods

### 2.1. Study design and participants

This was a single-center, retrospective observational study conducted at Shijiazhuang Eighth Hospital, Shijiazhuang, China. Patients who were clinically diagnosed with CD between January 2019 and December 2023 were screened for eligibility. According to the presence or absence of HPNCG, eligible patients were divided into a CD-alone group and a CD with HPNCG group. The study was conducted in accordance with the principles of the Declaration of Helsinki and reported according to the Strengthening the Reporting of Observational Studies in Epidemiology statement.

This study was approved by the Ethics Committee of Shijiazhuang Eighth Hospital (approval no. 2023-IRB-127). Because of the retrospective nature of the study and the use of anonymized clinical data, the requirement for written informed consent was waived by the Ethics Committee.

### 2.2. Inclusion and exclusion criteria

Patients were included if they met all of the following criteria: age ≥ 18 years; fulfillment of the clinical diagnostic criteria for CD; completion of upper gastrointestinal endoscopy with duodenal and/or gastric mucosal biopsy; clearly documented *H pylori* infection status; and relatively complete clinical data, including symptoms, laboratory findings, and histopathological results.

Patients were excluded if they met any of the following criteria: severe missing clinical data that precluded group classification or analysis of major variables; no endoscopic or histopathological examination; unclear *H pylori* infection status; gastrointestinal malignancy, severe infection, or other diseases that could substantially affect the histopathological assessment of the gastrointestinal mucosa; a history of gastric surgery or other specific conditions known to alter gastric mucosal structure; or recent treatment that might affect the assessment of *H pylori* status or gastric mucosal inflammatory changes.

### 2.3. Data collection and study variables

Clinical data were extracted from the hospital electronic medical record system. The collected information included demographic and baseline characteristics, clinical manifestations, comorbidities and related clinical conditions, laboratory findings, and endoscopic and histopathological variables. Demographic and baseline variables included sex, age, age group (18–29 years, 30–59 years, and ≥ 60 years), disease duration, body mass index, family history of autoimmune disease, smoking history, and alcohol consumption. Clinical manifestations included diarrhea, abdominal bloating, abdominal pain, nausea/vomiting, poor appetite, and weight loss. Comorbidities and related clinical conditions included anemia, hypoproteinemia, osteopenia or osteoporosis, liver function abnormality, Hashimoto thyroiditis, type 1 diabetes mellitus, and other autoimmune diseases. Laboratory findings included hematologic and nutritional parameters (hemoglobin, ferritin, albumin, total protein, folate, and vitamin B12), liver biochemical parameters (alanine aminotransferase, aspartate aminotransferase, and total bilirubin), and electrolyte and bone metabolism-related parameters (calcium, phosphorus, 25-hydroxyvitamin D, and alkaline phosphatase). Endoscopic and histopathological variables included duodenal Marsh grade, the degree of chronic inflammatory cell infiltration in the gastric mucosa, and the presence of glandular atrophy and intestinal metaplasia.

### 2.4. Diagnostic criteria and definitions

#### 2.4.1. Diagnostic criteria for CD

The diagnosis of CD was based on a comprehensive assessment of compatible clinical manifestations, CD-related serological findings, upper gastrointestinal endoscopy, and duodenal histopathology, in accordance with established clinical guidelines for the diagnosis and management of CD. Clinical manifestations mainly included diarrhea, abdominal bloating, weight loss, and anemia. Serological assessment included CD-related antibodies when available, and duodenal biopsy was used to evaluate characteristic mucosal changes, including villous atrophy, crypt hyperplasia, and increased intraepithelial lymphocytes. The final diagnosis was confirmed by gastroenterologists based on the overall clinical and pathological assessment.^[31–33]^

#### 2.4.2. Diagnostic criteria for HPNCG

HPNCG was defined as histopathological evidence of chronic gastritis in the absence of evidence of *H pylori* infection, after exclusion of *H pylori*-related gastritis.^[34,35]^

#### 2.4.3. Assessment of *H pylori* status

*H pylori* infection status was determined based on gastric mucosal histopathology, urea breath test results, and relevant medical records. The result closest to the time of endoscopic examination was used for classification.

#### 2.4.4. Duodenal histopathological grading

The severity of duodenal mucosal injury was assessed using the Marsh classification, which grades CD-related enteropathy according to intraepithelial lymphocytosis, crypt hyperplasia, and the degree of villous atrophy. For statistical analysis, Marsh grades were further categorized as I–IIIa and IIIb–IIIc to distinguish less severe mucosal injury from more advanced villous atrophy.^[36]^

#### 2.4.5. Definition of gastric histopathological variables

The degree of chronic inflammatory cell infiltration in the gastric antrum and/or body mucosa was recorded and classified as mild or moderate-to-severe according to the pathological report. The presence of glandular atrophy and intestinal metaplasia was also recorded.

#### 2.4.6. Definition of anemia

Anemia was defined according to hemoglobin levels in combination with the clinical diagnosis, based on institutional laboratory reference ranges or routine clinical criteria.

#### 2.4.7. Definition of hypoproteinemia

Hypoproteinemia was defined as a serum albumin level below the lower limit of the institutional reference range.

#### 2.4.8. Definition of osteopenia or osteoporosis

Osteopenia or osteoporosis was determined according to bone mineral density examination results and was analyzed as a binary variable.

#### 2.4.9. Definition of liver function abnormality

Liver function abnormality was defined as clinically significant abnormal liver biochemical findings during hospitalization or at the time of evaluation, or as clearly documented in the medical records.

#### 2.4.10. Definition of other autoimmune diseases

Hashimoto thyroiditis, type 1 diabetes mellitus, and other autoimmune diseases were recorded. Other autoimmune diseases were defined as autoimmune disorders other than Hashimoto thyroiditis and type 1 diabetes mellitus that were clearly documented in the medical records.

### 2.5. Quality control

Clinical data were independently extracted from the hospital electronic medical record system by 2 investigators and entered using a standardized data collection form. Key variables, including group classification, histopathological grading, and major laboratory findings, were checked to ensure data accuracy and consistency. The classification of duodenal and gastric mucosal pathology was based on formal pathological reports, and pathological information was extracted directly from the original reports. Doubtful cases were reviewed by a senior physician when necessary. Only patients with complete data for the major study variables were included, and cases with missing key information that precluded group classification or analysis of major variables were excluded before the final analysis.

### 2.6. Sample size

Because this was a retrospective single-center study, no formal a priori sample size calculation was performed. All eligible adult patients with CD who met the inclusion and exclusion criteria between January 2019 and December 2023 were consecutively included. Therefore, the final sample size was determined by the number of eligible cases available during the study period. Given the relatively small sample size, the regression and receiver operating characteristic (ROC) analyses were considered exploratory.

### 2.7. Statistical analysis

All statistical analyses were performed using SPSS version 26.0 (IBM Corp.). Continuous variables were first tested for normality. Normally distributed continuous variables were expressed as mean ± standard deviation and compared using the independent-samples *t* test, whereas non-normally distributed continuous variables were expressed as median (interquartile range) and compared using the Mann–Whitney *U* test. Categorical variables were presented as number (percentage) and compared using the chi-square test or Fisher exact test, as appropriate.

The presence of HPNCG was used as the dependent variable in logistic regression analyses. Univariate logistic regression analysis was first performed, including age, sex, abdominal bloating, anemia, osteopenia or osteoporosis, liver function abnormality, and Marsh grade IIIb–IIIc as candidate variables. Odds ratios (ORs) and 95% confidence intervals (CIs) were calculated. Variables with *P* < .05 in the univariate analysis were subsequently entered into the multivariate logistic regression model using the enter method, and adjusted ORs with 95% CIs were estimated.

ROC curve analysis was used to evaluate the discriminatory performance of Marsh grade IIIb–IIIc alone and the combined model for identifying CD complicated by HPNCG. The area under the curve (AUC), 95% CI, sensitivity, specificity, and Youden index were calculated. The combined model was constructed from the predicted probabilities generated by logistic regression. In addition, stratified analyses according to age, sex, disease duration, anemia status, and osteopenia/osteoporosis status were conducted to assess the consistency of the association between Marsh grade IIIb–IIIc and CD complicated by HPNCG across subgroups. Model stability was further evaluated using stepwise logistic regression. All statistical tests were two-sided, and *P* < .05 was considered statistically significant.

## 3. Results

### 3.1. Baseline characteristics of the study population

**Figure 1.**
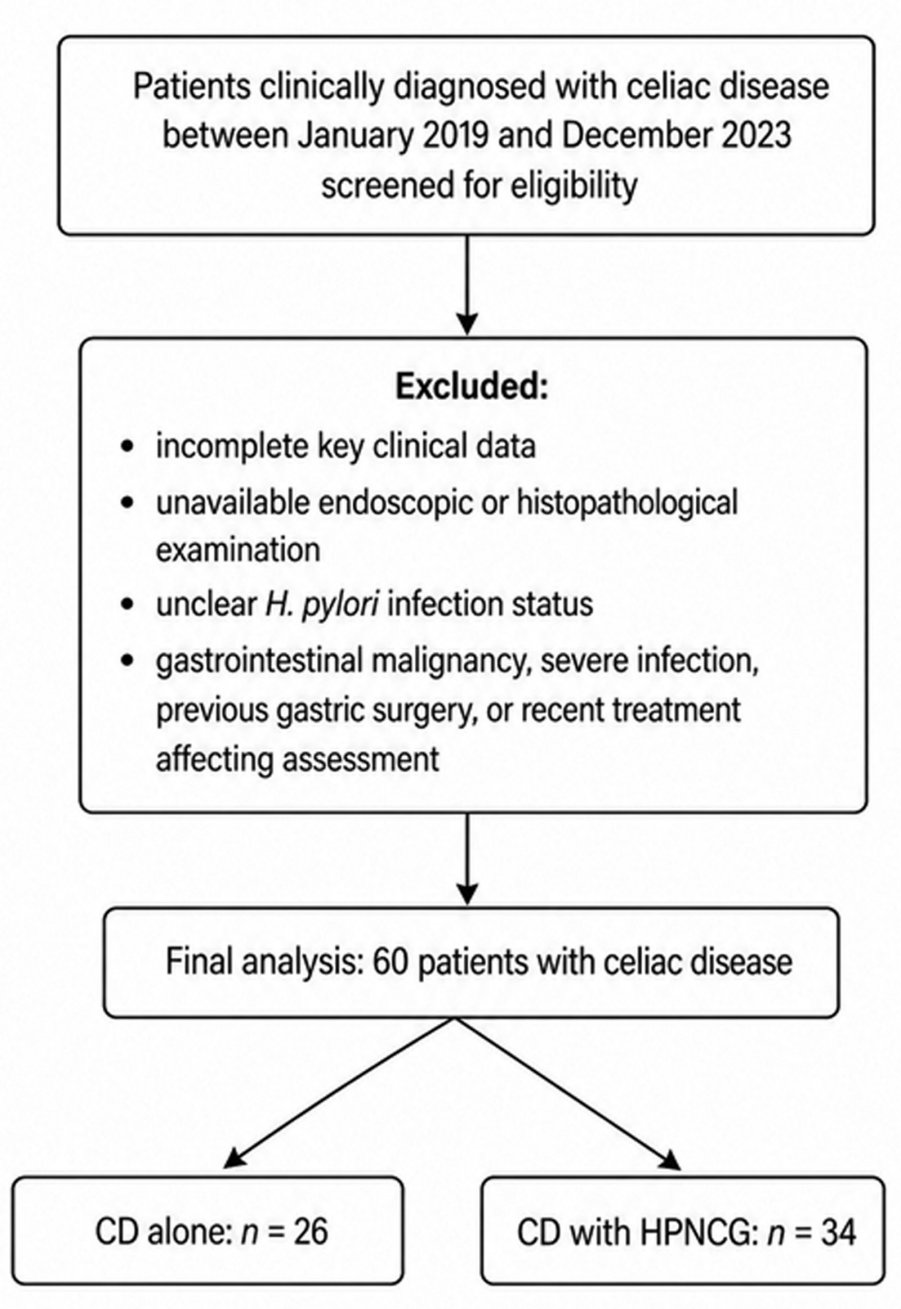
Flowchart of patient selection. Patients clinically diagnosed with celiac disease between January 2019 and December 2023 were screened for eligibility. After exclusion of patients with incomplete key clinical data, unavailable endoscopic or histopathological examination, unclear *H pylori* infection status, gastrointestinal malignancy, severe infection, previous gastric surgery, or recent treatment affecting assessment, 60 patients were included in the final analysis. Among them, 26 patients were classified as the CD-alone group and 34 as the CD with HPNCG group. CD = celiac disease, HPNCG = *Helicobacter pylori*-negative chronic gastritis.

After excluding patients with incomplete key clinical data, unavailable endoscopic or histopathological findings, unclear *H pylori* infection status, or other exclusion criteria, 60 patients were included in the final analysis (Fig. 1). A total of 60 patients with CD were included in the final analysis, including 26 patients in the CD-alone group and 34 patients in the CD with HPNCG group. Overall, 27 patients (45.00%) were male and 33 (55.00%) were female. The mean age was 41.82 ± 15.17 years, and the median disease duration was 1.80 (0.70–4.00) years. Most patients were aged 30–59 years (70.00%). As shown in Table 1, there were no statistically significant differences between the 2 groups in sex, age, age distribution, disease duration, body mass index, family history of autoimmune disease, smoking history, or alcohol consumption (all *P* > .05).

**Table 1:** Baseline demographic and clinical characteristics of patients with celiac disease according to the presence of *Helicobacter pylori*-negative chronic gastritis.

Variable	Total (n = 60)	CD alone (n = 26)	CD with HPNCG (n = 34)	*P* value
Sex, n (%)				.492
Male	27 (45.00)	13 (50.00)	14 (41.18)	
Female	33 (55.00)	13 (50.00)	20 (58.82)	
Age, yrs	41.82 ± 15.17	40.27 ± 14.68	43.00 ± 15.59	.497
Age group, n (%)				.808
18–29 yrs	8 (13.33)	4 (15.38)	4 (11.76)	
30–59 yrs	42 (70.00)	18 (69.23)	24 (70.59)	
≥60 yrs	10 (16.67)	4 (15.38)	6 (17.65)	
Disease duration, yrs	1.80 (0.70, 4.00)	1.60 (0.60, 3.50)	1.95 (0.80, 4.30)	.561
Body mass index, kg/m^2^	20.06 ± 2.89	20.34 ± 2.76	19.85 ± 2.98	.515
Family history of autoimmune disease, n (%)	9 (15.00)	3 (11.54)	6 (17.65)	.519
Smoking history, n (%)	11 (18.33)	5 (19.23)	6 (17.65)	.874
Alcohol consumption, n (%)	8 (13.33)	4 (15.38)	4 (11.76)	.684

Data are presented as mean ± standard deviation, median (interquartile range), or number (percentage), as appropriate. Comparisons between the 2 groups were performed using the independent-samples *t* test for normally distributed continuous variables, the Mann–Whitney *U* test for non-normally distributed continuous variables, and the chi-square test or Fisher exact test for categorical variables, as appropriate. A two-sided *P* < .05 was considered statistically significant.

CD = celiac disease, HPNCG = *Helicobacter pylori*-negative chronic gastritis.

### 3.2. Gastrointestinal manifestations

The frequencies of major gastrointestinal symptoms were compared between the 2 groups (Table 2). Patients with CD complicated by HPNCG had a significantly higher prevalence of abdominal bloating than those with CD alone (61.76% [21/34] vs 30.77% [8/26], *P* = .018). No significant between-group differences were observed for diarrhea (55.88% vs 46.15%, *P* = .458), abdominal pain (35.29% vs 23.08%, *P* = .308), nausea/vomiting (20.59% vs 11.54%, *P* = .347), poor appetite (32.35% vs 19.23%, *P* = .257), or weight loss (29.41% vs 23.08%, *P* = .586).

**Table 2:** Comparison of major gastrointestinal manifestations between patients with celiac disease alone and those with celiac disease complicated by *Helicobacter pylori*-negative chronic gastritis.

Symptom	CD alone (n = 26)	CD with HPNCG (n = 34)	*P* value
Diarrhea, n (%)	12 (46.15)	19 (55.88)	.458
Abdominal bloating, n (%)	8 (30.77)	21 (61.76)	.018
Abdominal pain, n (%)	6 (23.08)	12 (35.29)	.308
Nausea/vomiting, n (%)	3 (11.54)	7 (20.59)	.347
Poor appetite, n (%)	5 (19.23)	11 (32.35)	.257
Weight loss, n (%)	6 (23.08)	10 (29.41)	.586

Data are presented as number (percentage). Comparisons between groups were performed using the chi-square test or Fisher exact test, as appropriate. A two-sided *P* < .05 was considered statistically significant.

CD = celiac disease, HPNCG = *Helicobacter pylori*-negative chronic gastritis.

### 3.3. Comorbidities and related clinical conditions

As shown in Table 3, the prevalence of anemia was significantly higher in patients with CD complicated by HPNCG than in those with CD alone (58.82% [20/34] vs 26.92% [7/26], *P* = .013). Osteopenia or osteoporosis was also more common in the CD with HPNCG group (38.24% [13/34] vs 15.38% [4/26], *P* = .048). In addition, liver function abnormality occurred more frequently in patients with CD complicated by HPNCG than in those with CD alone (23.53% [8/34] vs 3.85% [1/26], *P* = .036). Although hypoproteinemia was more prevalent in the CD with HPNCG group (32.35% vs 15.38%), the difference did not reach statistical significance (*P* = .129). No significant between-group differences were observed in the prevalence of Hashimoto thyroiditis, type 1 diabetes mellitus, or other autoimmune diseases (all *P* > .05).

**Table 3:** Comparison of comorbidities and related clinical conditions between patients with celiac disease alone and those with celiac disease complicated by *Helicobacter pylori*-negative chronic gastritis.

Variable	CD alone (n = 26)	CD with HPNCG (n = 34)	*P* value
Anemia, n (%)	7 (26.92)	20 (58.82)	.013
Hypoproteinemia, n (%)	4 (15.38)	11 (32.35)	.129
Osteopenia or osteoporosis, n (%)	4 (15.38)	13 (38.24)	.048
Liver function abnormality, n (%)	1 (3.85)	8 (23.53)	.036
Hashimoto thyroiditis, n (%)	3 (11.54)	5 (14.71)	.719
Type 1 diabetes mellitus, n (%)	1 (3.85)	2 (5.88)	.719
Other autoimmune diseases, n (%)	2 (7.69)	4 (11.76)	.602

Comparisons between groups were performed using the chi-square test or Fisher exact test, as appropriate. Liver function abnormality was defined as the presence of clinically significant abnormal liver biochemical parameters during hospitalization or at the time of evaluation. Other autoimmune diseases included autoimmune disorders other than Hashimoto thyroiditis and type 1 diabetes mellitus. A two-sided *P* < .05 was considered statistically significant.

CD = celiac disease, HPNCG = *Helicobacter pylori*-negative chronic gastritis.

### 3.4. Laboratory findings

The comparison of major laboratory parameters between the 2 groups is shown in Table 4. Patients with CD complicated by HPNCG had significantly lower hemoglobin levels than those with CD alone (108.57 ± 19.41 g/L vs 119.84 ± 17.63 g/L, *P* = .024). Ferritin levels were also lower in the CD with HPNCG group (18.50 [9.20–31.60] μg/L vs 27.80 [13.50–42.40] μg/L, *P* = .041). Albumin levels tended to be lower in patients with CD complicated by HPNCG, although the difference did not reach statistical significance (36.93 ± 5.17 g/L vs 39.12 ± 4.68 g/L, *P* = .087). With respect to liver biochemical parameters, aspartate aminotransferase levels were significantly higher in the CD with HPNCG group (34.21 [23.03–48.47] U/L vs 24.58 [18.47–31.84] U/L, *P* = .032), whereas alanine aminotransferase levels showed no statistically significant difference between groups (32.76 [22.48–44.63] U/L vs 27.41 [18.92–36.84] U/L, *P* = .096). Among electrolyte and bone metabolism-related parameters, serum calcium levels were comparable between the 2 groups (2.16 ± 0.18 mmol/L vs 2.21 ± 0.15 mmol/L, *P* = .271), whereas 25-hydroxyvitamin D levels were significantly lower in the CD with HPNCG group (14.30 [9.80–19.70] ng/mL vs 19.60 [13.20–24.10] ng/mL, *P* = .038).

**Table 4:** Comparison of major laboratory parameters between patients with CD alone and those with CD complicated by HPNCG.

Variable	CD alone (n = 26)	CD with HPNCG (n = 34)	*P* value
Hematologic and nutritional parameters			
Hemoglobin, g/L	119.84 ± 17.63	108.57 ± 19.41	.024
Ferritin, μg/L	27.80 (13.50–42.40)	18.50 (9.20–31.60)	.041
Albumin, g/L	39.12 ± 4.68	36.93 ± 5.17	.087
Total protein, g/L	66.84 ± 6.25	64.11 ± 7.03	.121
Folate, ng/mL	7.94 (5.86–10.43)	6.71 (4.52–8.94)	.146
Vitamin B12, pg/mL	326.45 ± 102.37	301.18 ± 96.54	.336
Liver biochemical parameters			
ALT, U/L	27.41 (18.92–36.84)	32.76 (22.48–44.63)	.096
AST, U/L	24.58 (18.47–31.84)	34.21 (23.03–48.47)	.032
Total bilirubin, μmol/L	12.36 ± 4.17	13.58 ± 4.89	.314
Electrolyte and bone metabolism-related parameters			
Calcium, mmol/L	2.21 ± 0.15	2.16 ± 0.18	.271
Phosphorus, mmol/L	1.13 ± 0.18	1.08 ± 0.21	.327
25-Hydroxyvitamin D, ng/mL	19.60 (13.20–24.10)	14.30 (9.80–19.70)	.038
Alkaline phosphatase, U/L	87.46 (69.35–104.28)	95.72 (76.44–118.36)	.183

Data are presented as mean ± standard deviation or median (interquartile range), as appropriate. Comparisons between groups were performed using the independent-samples *t* test for normally distributed variables and the Mann–Whitney *U* test for non-normally distributed variables, as appropriate. A two-sided *P* < .05 was considered statistically significant.

ALT = alanine aminotransferase, AST = aspartate aminotransferase, CD = celiac disease, HPNCG = *Helicobacter pylori*-negative chronic gastritis.

### 3.5. Endoscopic and histopathological findings

The comparison of endoscopic and histopathological characteristics between the 2 groups is presented in Table 5. Patients with CD complicated by HPNCG had a significantly higher proportion of severe duodenal mucosal injury, with Marsh grade IIIb–IIIc observed in 88.24% (30/34) of patients, compared with 61.54% (16/26) in the CD-alone group (*P* = .019). Regarding gastric mucosal pathology, moderate-to-severe chronic inflammatory cell infiltration in the gastric antrum and/or body was more common in the CD with HPNCG group than in the CD-alone group (64.71% [22/34] vs 34.62% [9/26], *P* = .022). In contrast, no significant between-group differences were found in the prevalence of glandular atrophy (20.59% vs 15.38%, *P* = .606) or intestinal metaplasia (11.76% vs 7.69%, *P* = .602).

**Table 5:** Comparison of endoscopic and histopathological characteristics between patients with celiac disease alone and those with celiac disease complicated by *Helicobacter pylori*-negative chronic gastritis.

Variable	CD alone (n = 26)	CD with HPNCG (n = 34)	*P* value
Duodenal histopathology			
Marsh grade I–IIIa, n (%)	10 (38.46)	4 (11.76)	.019
Marsh grade IIIb–IIIc, n (%)	16 (61.54)	30 (88.24)	.019
Gastric mucosal histopathology			
Mild chronic inflammatory cell infiltration, n (%)	17 (65.38)	12 (35.29)	.022
Moderate-to-severe chronic inflammatory cell infiltration, n (%)	9 (34.62)	22 (64.71)	.022
Glandular atrophy, n (%)	4 (15.38)	7 (20.59)	.606
Intestinal metaplasia, n (%)	2 (7.69)	4 (11.76)	.602

Comparisons between groups were performed using the chi-square test or Fisher exact test, as appropriate. Marsh grade was classified according to the histopathological severity of duodenal mucosal injury. For regression analysis, Marsh grade was dichotomized as I–IIIa versus IIIb–IIIc. Moderate-to-severe chronic inflammatory cell infiltration refers to histological evidence of moderate or marked chronic inflammatory infiltrates in the gastric antrum and/or body mucosa. A two-sided *P* < .05 was considered statistically significant.

CD = celiac disease, HPNCG = *Helicobacter pylori*-negative chronic gastritis.

### 3.6. Logistic regression analyses of factors associated with CD complicated by HPNCG

Abdominal bloating was associated with a 3.32-fold increased odds of HPNCG in patients with CD (OR = 3.32, 95% CI: 1.14–9.67, *P* = .028). Anemia also showed a significant association with the presence of HPNCG (OR = 3.67, 95% CI: 1.24–10.88, *P* = .019), as did osteopenia/osteoporosis (OR = 3.21, 95% CI: 1.05–9.79, *P* = .041). Furthermore, patients with Marsh grade IIIb–IIIc exhibited significantly higher odds of HPNCG compared to those with Marsh grade I–IIIa (OR = 4.39, 95% CI: 1.32–14.58, *P* = .016). Variables with *P* < .05 in the univariate analysis were subsequently included in the multivariate logistic regression model. After adjustment, Marsh grade IIIb–IIIc remained the sole factor independently associated with CD complicated by HPNCG (OR = 3.96, 95% CI: 1.05–14.91, *P* = .042; Table 6).

**Table 6:** Univariate and multivariate logistic regression analyses of factors associated with celiac disease complicated by *Helicobacter pylori*-negative chronic gastritis.

Variable	Univariate OR (95% CI)	*P* value	Multivariate OR (95% CI)	*P* value
Age, yrs	1.02 (0.99–1.06)	.214	–	–
Female sex	1.43 (0.50–4.08)	.502	–	–
Abdominal bloating	3.32 (1.14–9.67)	.028	2.26 (0.70–7.24)	.171
Anemia	3.67 (1.24–10.88)	.019	2.34 (0.70–7.78)	.166
Osteopenia/osteoporosis	3.21 (1.05–9.79)	.041	2.11 (0.54–8.19)	.284
Liver function abnormality	6.24 (0.78–49.93)	.086	–	–
**Marsh grade IIIb–IIIc**	**4.39 (1.32–14.58**)	**.016**	**3.96 (1.05–14.91**)	**.042**

The dependent variable was the presence of HPNCG in patients with CD. Variables with *P* < .05 in the univariate analysis were entered into the multivariate logistic regression model. Marsh grade was dichotomized as I–IIIa versus IIIb–IIIc. A two-sided *P* < .05 was considered statistically significant. Statistically significant values are shown in bold. “–” indicates that the variable was not included in the multivariate model.

CD = celiac disease, CI = confidence interval, HPNCG = *Helicobacter pylori*-negative chronic gastritis, OR = odds ratio.

### 3.7. ROC analysis for identifying CD complicated by HPNCG

ROC curve analysis was performed to evaluate the discriminatory performance of Marsh grade IIIb–IIIc and the combined logistic regression model for identifying CD complicated by HPNCG (Table 7 and Fig. S1, Supplemental Digital Content). Marsh grade IIIb–IIIc alone yielded an AUC of 0.73 (95% CI: 0.60–0.85, *P* = .006). The combined model incorporating abdominal bloating, anemia, osteopenia/osteoporosis, and Marsh grade IIIb–IIIc showed a better discriminatory ability, with an AUC of 0.81 (95% CI: 0.69–0.91, *P* < .001), a sensitivity of 76.47%, a specificity of 73.08%, and a Youden index of 0.50.

**Table 7:** ROC analysis of Marsh grade IIIb–IIIc and the combined model for identifying CD complicated by *Helicobacter pylori*-negative chronic gastritis.

Model	AUC (95% CI)	*P* value	Sensitivity, %	Specificity, %	Youden index
Marsh grade IIIb–IIIc	0.73 (0.60–0.85)	.006	88.24	38.46	0.27
Combined model	0.81 (0.69–0.91)	<.001	76.47	73.08	0.50

The combined model was derived from logistic regression including abdominal bloating, anemia, osteopenia/osteoporosis, and Marsh grade IIIb–IIIc. A two-sided *P* < .05 was considered statistically significant.

AUC = area under the curve, CD = celiac disease, CI = confidence interval, ROC = receiver operating characteristic.

### 3.8. Sensitivity and supplementary analyses

To evaluate the consistency of the observed association between Marsh grade IIIb–IIIc and HPNCG in patients with CD, stratified analyses were performed across prespecified clinical subgroups (Table S1, Supplemental Digital Content). The positive association remained directionally consistent in all subgroups examined, including those defined by age, sex, disease duration, anemia, and osteopenia/osteoporosis, suggesting no significant effect modification. In addition, stepwise logistic regression confirmed the stability of the finding, with Marsh grade IIIb–IIIc retained as an independent factor in the final model (Table S2, Supplemental Digital Content).

## 4. Discussion

In this study, we compared the clinical and histopathological characteristics of patients with CD alone and those with CD complicated by HPNCG. The results showed that patients with CD complicated by HPNCG had a heavier clinical burden, as reflected by a higher prevalence of abdominal bloating, anemia, osteopenia or osteoporosis, and liver function abnormality. Histopathological analysis further showed that severe duodenal villous atrophy, represented by Marsh grade IIIb–IIIc, and more pronounced chronic inflammatory cell infiltration of the gastric mucosa were more common in this group. In addition, multivariate logistic regression identified Marsh grade IIIb–IIIc as the only independent factor associated with CD complicated by HPNCG. Taken together, these findings suggest that CD complicated by HPNCG may represent a subgroup characterized by more extensive gastrointestinal mucosal injury and a greater overall clinical burden.

Abdominal bloating was more common in patients with CD complicated by HPNCG and represented one of the most notable symptomatic differences between the 2 groups. Although bloating is not a specific manifestation of CD, it may be more clinically informative when gastric involvement is present. This finding may be related to several factors. Villous atrophy in the small intestine can impair nutrient absorption and promote abnormal intraluminal fermentation, both of which may contribute to bloating.^[37–40]^ At the same time, chronic gastric mucosal inflammation may affect gastric motility, gastric emptying, and upper gastrointestinal comfort.^[41,42]^ When both the stomach and small intestine are involved, postprandial fullness and upper abdominal discomfort may become more pronounced.^[43,44]^ These observations underscore the need for heightened clinical awareness of gastric involvement in patients with CD who report significant bloating, particularly when accompanied by severe villous atrophy or evidence of nutritional compromise. The findings further suggest that a comprehensive evaluation of CD should encompass not only the degree of enteropathy but also associated systemic and nutritional consequences.

The higher prevalence of anemia in patients with CD complicated by HPNCG, along with lower hemoglobin and ferritin levels, points to a greater burden of chronic malabsorption in this subgroup. CD itself impairs the absorption of iron, folate, and other nutrients due to reduced small intestinal absorptive surface area.^[45–47]^ Concurrent chronic gastric inflammation may further disrupt iron metabolism by altering gastric acidity and the intragastric environment.^[48,49]^ In addition, inflammation-related pathways may contribute to reduced hemoglobin levels.^[50,51]^ Although hypoproteinemia and lower albumin levels did not reach statistical significance consistently, the overall pattern supports poorer nutritional status and suggests that the functional impairment extends beyond single nutritional markers.^[52–54]^ Additionally, osteopenia or osteoporosis was more common in the HPNCG group, and 25-hydroxyvitamin D levels were lower. These findings align with the known nutritional consequences of CD and may reflect a greater overall disease burden in patients with HPNCG.

The most important finding of this study is that Marsh grade IIIb–IIIc was more frequent in patients with CD complicated by HPNCG and remained significant in the multivariate logistic regression model, indicating a stable association between severe duodenal mucosal injury and the presence of HPNCG in CD. Marsh grade IIIb–IIIc reflects more advanced villous atrophy and more severe small intestinal mucosal damage.^[55]^ This degree of pathological change may also indicate stronger immune activation, more substantial malabsorption, and a broader inflammatory background affecting the gastrointestinal mucosa.^[18,56]^ ROC analysis further validated the utility of Marsh grade IIIb–IIIc, which alone showed acceptable discriminatory performance and contributed to a higher AUC in the combined model. This underscores that when severe villous atrophy is present in CD, clinicians should maintain a heightened awareness of potential gastric involvement, especially HPNCG, and consider a more thorough assessment of gastric mucosal changes.

Patients with CD complicated by HPNCG exhibited a significantly higher degree of moderate-to-severe chronic inflammatory infiltration in the gastric mucosa, supporting the notion of a broader inflammatory diathesis along the gastrointestinal tract in this population. In contrast, the lack of significant between-group differences in glandular atrophy or intestinal metaplasia indicates that the gastric pathology associated with HPNCG is predominantly inflammatory in nature, rather than being driven by atrophic or metaplastic changes. The findings of this study are consistent with previous reports suggesting that patients with CD may present with gastric mucosal inflammation and *H pylori*-negative gastritis.^[21,57–61]^ The present results extend this observation by showing that CD complicated by HPNCG is not only associated with more evident gastric inflammatory changes, but also with a heavier burden of gastrointestinal symptoms, nutritional abnormalities, and more severe duodenal mucosal injury.

Clinically, the presence of prominent bloating, anemia, osteopenia/osteoporosis, or severe villous atrophy in patients with CD should raise suspicion for HPNCG. Such patients may benefit from more comprehensive evaluation, including gastric mucosal assessment, and require closer attention to nutritional status, bone health, and liver function during follow-up.

Several limitations of this study should be acknowledged. The study was conducted at a single center with a retrospective observational design, which may have introduced selection bias and information bias. This design also limits the ability to infer causal relationships between CD and HPNCG. Therefore, the multivariate regression and ROC analyses should be interpreted as exploratory rather than as the development of a definitive prediction model. External validation in larger independent cohorts is needed before the combined model can be applied in clinical practice. In addition, the sample size was relatively small, with only 60 patients included in the final analysis. The limited number of events for some variables may have reduced the precision of the regression estimates, as reflected by the relatively wide CIs. Another issue is the assessment of *H pylori* status. If different diagnostic methods were used across patients rather than a fully standardized approach, some degree of misclassification cannot be excluded. The range of variables available for analysis was also limited. More detailed serological and immunological data related to CD, as well as long-term follow-up information, were not included in the present study. As a result, the underlying mechanisms linking CD and HPNCG could not be further explored. Further studies with multicenter and larger sample sizes are needed to validate the clinical and histopathological features of CD complicated by HPNCG. Prospective follow-up studies are also warranted to clarify the impact of HPNCG on treatment response, nutritional recovery, and long-term outcomes in patients with CD.

## 5. Conclusions

Compared with patients with CD alone, those complicated by HPNCG exhibited a higher prevalence of abdominal bloating, anemia, osteopenia/osteoporosis, and liver function abnormalities. Histopathologically, this subgroup demonstrated more severe duodenal villous atrophy and more pronounced chronic inflammatory infiltration in the gastric mucosa, suggesting more extensive gastrointestinal mucosal involvement. Marsh grade IIIb–IIIc was independently associated with the presence of HPNCG in patients with CD. Therefore, the multivariate regression and ROC analyses should be interpreted as exploratory rather than as the development of a definitive prediction model. External validation in larger independent cohorts is needed before the combined model can be applied in clinical practice.

## Acknowledgments

The authors sincerely thank all study participants for their invaluable contributions.

## Author contributions

**Conceptualization:** Nan Gu, Li Ma, Lei Zhang, Fei-Ran Jia, Xiao-Yun Zhou, Yue Wang.

**Writing – original draft:** Li Ma.

**Writing – review & editing:** Li Ma.





**Figure:**