# Supplementary material for: Clinical characteristics and associated factors of celiac disease complicated by Helicobacter pylori-negative chronic gastritis: A retrospective study
Source: Medicine (Baltimore). 2026 May 29;105(22):e48950. doi: 10.1097/MD.0000000000048950 (PMC13225508; doi:10.1097/MD.0000000000048950)
Supplement: Supplementary file 1 [file medi-105-e48950-s002.docx]

**Supplementary Table S1. Stratified analyses of the association between Marsh grade IIIb–IIIc and celiac disease complicated by Helicobacter pylori-negative chronic gastritis**

| **Stratification variable** | **Subgroup** | **OR (95% CI)** | **P value** |
| --- | --- | --- | --- |
| **Age** | <40 years | 3.78 (0.81–17.56) | 0.091 |
|  | ≥40 years | 4.26 (1.03–17.63) | 0.046 |
| **Sex** | Male | 3.94 (0.72–21.47) | 0.113 |
|  | Female | 4.18 (1.01–17.28) | 0.048 |
| **Disease duration** | <2 years | 3.67 (0.84–15.96) | 0.083 |
|  | ≥2 years | 4.52 (1.07–19.05) | 0.040 |
| **Anemia** | No | 3.21 (0.77–13.36) | 0.108 |
|  | Yes | 4.47 (1.02–19.61) | 0.047 |
| **Osteopenia/osteoporosis** | No | 3.86 (1.04–14.31) | 0.043 |
|  | Yes | 4.23 (0.68–26.35) | 0.124 |

**Abbreviations:** OR, odds ratio; CI, confidence interval; CD, celiac disease; HPNCG, *Helicobacter pylori*-negative chronic gastritis.
Stratified logistic regression analyses were performed to assess whether the association between Marsh grade IIIb–IIIc and CD complicated by HPNCG remained consistent across clinically relevant subgroups.
Age was stratified as <40 years and ≥40 years; disease duration was stratified as <2 years and ≥2 years.
Marsh grade was dichotomized as I–IIIa versus IIIb–IIIc.
A two-sided *P* < 0.05 was considered statistically significant.
The relatively wide confidence intervals in some subgroups may reflect the limited sample size and reduced number of events within each stratum.
