# Supplementary material for: Clinical characteristics and associated factors of celiac disease complicated by Helicobacter pylori-negative chronic gastritis: A retrospective study
Source: Medicine (Baltimore). 2026 May 29;105(22):e48950. doi: 10.1097/MD.0000000000048950 (PMC13225508; doi:10.1097/MD.0000000000048950)
Supplement: Supplementary file 2 [file medi-105-e48950-s003.docx]

**Supplementary Table S2. Sensitivity analysis of the multivariate logistic regression model**

| **Model** | **Variables retained in final model** | **Adjusted OR (95% CI) for Marsh grade IIIb–IIIc** | **P value** |
| --- | --- | --- | --- |
| Primary multivariate model | Abdominal bloating, anemia, osteopenia/osteoporosis, Marsh grade IIIb–IIIc | 3.96 (1.05–14.91) | 0.042 |
| Stepwise multivariate model | Marsh grade IIIb–IIIc | 4.08 (1.11–15.02) | 0.035 |

The primary multivariate model included variables with *P* < 0.05 in the univariate analysis.
A stepwise logistic regression procedure was additionally performed to assess model stability.
The persistence of Marsh grade IIIb–IIIc in the final model suggests that the association was relatively robust despite the limited sample size.
